# Empowering Out‐of‐Home Participation and Collaborative Care in Early‐Stage Dementia: From Concept to Technology Use

**DOI:** 10.1002/alz70858_102455

**Published:** 2025-12-25

**Authors:** Amy Hwang, Camille Normandin, Rosalie H. Wang, Maxime Lussier, Habib Chaudhury, Sayeh Bayat, Ishaan Singla, Ron Beleno, Thomas Tannou

**Affiliations:** ^1^ Centre de recherche de l'Institut universitaire de gériatrie de Montréal, Montreal, QC, Canada; ^2^ University of Toronto, Toronto, ON, Canada; ^3^ Centre de Recherche de l'Institut Universitaire de Gériatrie de Montréal (CRIUGM), Montreal, QC, Canada; ^4^ Simon Fraser University, Vancouver, BC, Canada; ^5^ Hotchkiss Brain Institute, Calgary, AB, Canada; ^6^ WeTraq Inc, Whitby, ON, Canada; ^7^ RB33, Toronto, ON, Canada; ^8^ CRIUGM, CIUSS Centre‐Sud de l'Ile de Montréal, Montreal, QC, Canada

## Abstract

**Background:**

Remaining active and engaged in activities outside the home is crucial for preserving health, well‐being, and social participation as people age. However, person living with dementia (PLWD), including those with early‐stage cognitive impairment, often reduce their out‐of‐home participation—such as using public spaces, transportation, or attending social gatherings—due to barriers like accessibility challenges, fear, or safety concerns raised by their care partners. Current technological solutions have largely focused on ensuring safety of PLWD through tracking and locating features, but these approaches may inadvertently undermine their self‐management strategies, disregard their values, and exclude them from key decision‐making processes around risk‐taking, privacy, and care. There is a critical need for digital health innovations to prioritize ethical collaboration, supported decision‐making with PLWD, and promote PLWD's autonomy and dignity while supporting care relationships. Developing interventions based on nuanced understanding of the relational dynamics between PLWD and their care partners, particularly how they negotiate care strategies and situate technology in ways that mediate value‐driven conflicts

**Method:**

Using an expanded development phase of the complex interventions development framework, this study describes the systematic design of a smart insole intervention that aims to empower PLWD in their out‐of‐home participation and facilitate collaborative care practices with care partners.

**Results:**

The study presents a comprehensive example of complex intervention design, integrating co‐created insights from knowledge users with relevant theory and evidence. Key outputs include intervention principles that prioritize empowerment and cognitive accessibility for PLWD, hypothesized mechanisms of processes (e.g., dyadic care negotiation dynamics, self‐perception adjustment and technology adoption), and projected outcomes (e.g., enhanced out‐of‐home participation and improved dyadic care collaboration). Illustrative case studies further demonstrate potential real‐world applications of, and hypothesized outcomes resulting from, the smart insole intervention compared to current care practices

**Conclusion:**

A structured, evidence‐based approach to complex intervention design can support the translation of theory, user needs, and empirical evidence into actionable intervention strategies and evaluative frameworks. Digital health technologies, such as the smart insole intervention described here, hold promise for fostering empowerment and dignity among PLWD while promoting ethical, collaborative care arrangements with their care partners.

